# Fractionation of Bioactive Polysaccharides from *Volvariella volvacea* and Their Ameliorative Effects on Hepatic Lipid Accumulation

**DOI:** 10.3390/ijms27219750

**Published:** 2026-10-30

**Authors:** Wenbo Xia, Ruohan Zhao, Shuying Li

**Affiliations:** 1 School of Pharmaceutical Sciences, Zhejiang Chinese Medical University, Hangzhou 310053, China; 2 School of Pharmaceutical Sciences, Nanjing University of Chinese Medicine, Nanjing 210023, China

**Keywords:** *Volvariella volvacea*, polysaccharides, fractionation, hepatic lipid accumulation, mitochondrial function

## Abstract

*Volvariella volvacea* is a widely consumed edible mushroom, yet the lipid-lowering potential of its polysaccharides remains poorly understood. In this study, two polysaccharide-enriched fractions, VVP-1 and VVP-2, were isolated from *V. volvacea* fruiting bodies, with weight-average molecular weights of 13,789 and 17,829 Da, respectively. Their lipid-lowering effects were evaluated in palmitic acid (PA)-challenged AML12 mouse hepatocytes and in high-fat diet (HFD)-induced obese male C57BL/6J mice. Both fractions reduced PA-induced intracellular triglyceride (TG) levels in a dose-dependent manner, with VVP-2 showing a trend of greater efficacy. At the molecular level, VVP-2 exposure was associated with downregulation of the lipogenic genes *Acaca* and *Srebf1*, upregulation of the fatty acid oxidation-related genes *Cpt1a* and *Ppara*, decreased secretion of TNF-α, IL-6, and IL-1β, preservation of mitochondrial signal, restoration of mitochondrial membrane potential, and enhanced ATP production. In HFD-fed mice, VVP-2 (100 mg/kg) attenuated body-weight gain by 38.9%, reduced liver weight by 28.9% and epididymal, inguinal, and perirenal fat weights by 50.3%, 47.5%, and 28.8%, respectively, improved the serum lipid profile (TG reduced by 39.1%, TC by 33.5%, NEFA by 56.2%, and LDL-C by 56.8%), and alleviated hepatic lipid accumulation and liver injury. These findings suggest that VVP-2 may improve mitochondrial function and modulate lipid metabolism-related gene expression, potentially reducing lipid accumulation and inflammation in hepatocytes. These results support the potential of VVP-2 as a lipid-lowering functional food ingredient for the prevention of metabolic dysfunction-associated steatotic liver disease.

## 1. Introduction

Metabolic dysfunction-associated steatotic liver disease (MASLD), formerly known as nonalcoholic fatty liver disease (NAFLD), is one of the most common chronic liver diseases worldwide [1]. Its global prevalence is approximately 38% and continues to rise [2]. MASLD is characterized primarily by abnormal triglyceride (TG) accumulation in hepatocytes and may progress from simple steatosis to metabolic dysfunction-associated steatohepatitis (MASH), liver fibrosis, cirrhosis, and hepatocellular carcinoma [3,4]. Dysregulated hepatic lipid metabolism is a central driver of MASLD. It is manifested mainly by increased fatty acid uptake, enhanced de novo lipogenesis, impaired fatty acid β-oxidation, and reduced very-low-density lipoprotein secretion, ultimately causing excessive lipid deposition in hepatocytes [5]. Current management relies mainly on lifestyle intervention and lipid-lowering drugs, including statins and thiazolidinediones [6]. Recently, resmetirom and semaglutide have been approved for the treatment of noncirrhotic MASH with moderate-to-advanced fibrosis, representing the first pharmacological therapies for this condition [7,8,9]. Despite these advances, safe and effective lipid-lowering agents derived from natural products continue to attract increasing interest.

Polysaccharides are natural macromolecules widely distributed in plants, animals, and microorganisms and possess immunomodulatory, antitumor, antioxidant, hypoglycemic, and lipid-lowering activities [10,11]. Growing evidence indicates that fungal polysaccharides offer distinct benefits in ameliorating disorders of lipid metabolism. For example, *Pleurotus citrinopileatus* polysaccharides reduce lipid accumulation by improving the gut microbiota and chronic inflammation [12]. *Morchella esculenta* polysaccharides promote L-arginine biosynthesis and subsequently activate the AMPK/Sirt1/PGC-1α signaling axis, thereby enhancing fatty acid oxidation and improving lipid metabolic dysfunction [13]. *Cordyceps militaris* polysaccharides restore intestinal health and reduce blood glucose and lipid levels by increasing short-chain fatty acid levels, modulating the gut microbial community, and repairing intestinal tight junctions [14]. These findings highlight the considerable potential of edible mushroom polysaccharides as natural lipid-lowering functional ingredients.

*Volvariella volvacea* is one of the major cultivated edible mushrooms in China. Its annual production in China is estimated at 330,000 metric tons, accounting for 80% of global output. It is widely consumed because of its appealing flavor and rich nutritional value [15,16]. The mushroom is rich in protein, dietary fiber, vitamins, and minerals, and modern studies have demonstrated antioxidant, antitumor, immunomodulatory, and anti-inflammatory activities [17,18,19]. *V. volvacea* polysaccharides (VVPs) are among its principal active constituents and have demonstrated immunomodulatory and antioxidant activities [20,21]. However, their lipid-lowering effects remain poorly studied. Only a few reports have described inhibition of fat accumulation in *Caenorhabditis elegans* by VVPs [22], and the active fractions, molecular mechanisms, and dose–response relationships require systematic investigation.

The aim of this study was to isolate and partially characterize polysaccharide fractions from *V. volvacea* and to evaluate their lipid-lowering effects and potential molecular changes. Crude polysaccharides were obtained by hot-water extraction and ethanol precipitation, deproteinized, and sequentially fractionated by DEAE-52 cellulose and Sephadex G-100 chromatography. The resulting fractions were characterized for molecular weight and basic chemical composition, including total carbohydrate, uronic acid, and residual protein contents. Their lipid-lowering effects were then assessed in palmitic acid (PA)-challenged AML12 hepatocytes and in high-fat diet (HFD)-induced obese C57BL/6J mice, with simvastatin (SIM) as a positive control. Potential molecular changes were explored by RT-qPCR, transcriptomic profiling, and mitochondrial functional assays. This study aimed to provide a basis for developing *V. volvacea* polysaccharides as lipid-lowering functional food ingredients.

## 2. Results

### 2.1. Polysaccharide Yield and Protein Content

Approximately 45 g of crude polysaccharides were obtained from 500 g of dried *V. volvacea*, corresponding to an extraction yield of approximately 9%. The crude polysaccharides were deproteinized with trichloroacetic acid (TCA), and protein content was determined by the bicinchoninic acid (BCA) method. Deproteinization reduced protein content significantly from 21.0% to 2.5%, equivalent to a protein removal rate of approximately 88.1%. The polysaccharide retention rate after TCA treatment was 92.5% ± 1.5% (*n* = 3), indicating that the TCA method removed most free proteins while retaining the majority of the crude polysaccharides.

### 2.2. Fractionation of Crude Polysaccharides

**Figure 1:**
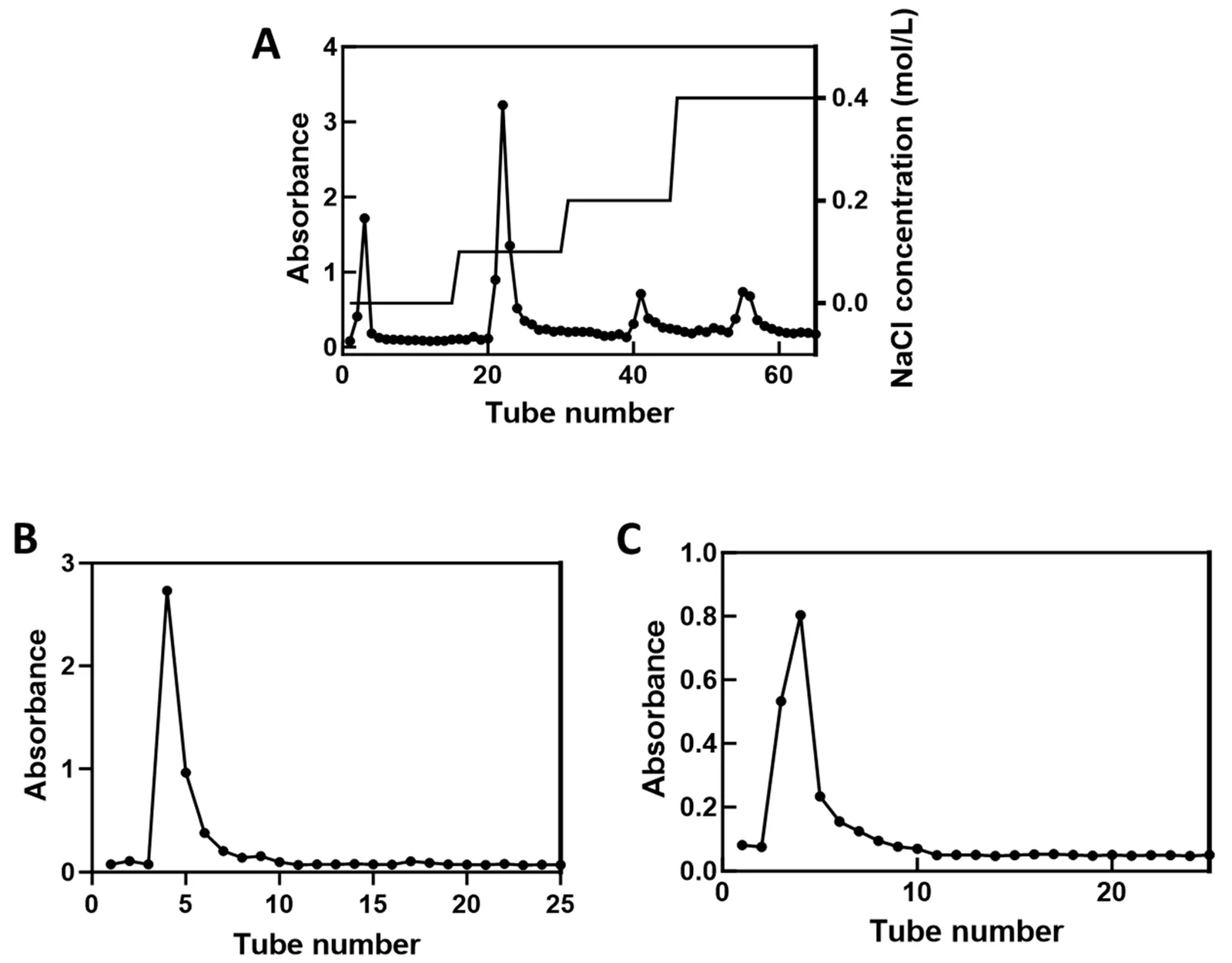
Fractionation of polysaccharides and VVP fractions by column chromatography. (**A**) DEAE-52 cellulose column chromatography. (**B**) Sephadex G-100 column chromatography (water-eluted fraction). (**C**) Sephadex G-100 column chromatography (0.1 mol/L NaCl-eluted fraction).

The deproteinized crude polysaccharides were separated on a DEAE-52 cellulose column and eluted sequentially with 0, 0.1, 0.2, and 0.4 mol/L NaCl. The polysaccharide content of each tube was measured using the phenol-sulfuric acid method, and the resulting elution profile is shown in Figure 1A. Most polysaccharides were recovered in the water-eluted peak and the 0.1 mol/L NaCl-eluted peak; both contained substantially more polysaccharide than the other peaks.

The principal DEAE-52 fractions, namely the water-eluted fraction and the 0.1 mol/L NaCl-eluted fraction, were separately fractionated further on Sephadex G-100 with distilled water as the mobile phase. Elution was monitored using the phenol-sulfuric acid method. As shown in Figure 1B, the water-eluted fraction produced a single symmetrical peak after Sephadex G-100 chromatography and was designated VVP-1. Similarly, the 0.1 mol/L NaCl-eluted fraction yielded one predominant symmetrical peak (Figure 1C) and was designated VVP-2.

The chemical compositions of the VVP-1 and VVP-2 fractions were analyzed. VVP-1 contained 95.32 ± 2.31% total carbohydrate, 3.72 ± 0.043% uronic acid and 0.11 ± 0.002% residual protein, whereas VVP-2 contained 95.66 ± 2.02% total carbohydrate, 4.03 ± 0.038% uronic acid and 0.10 ± 0.004% residual protein. Endotoxin levels were below 0.01 EU/mg in both fractions, excluding LPS contamination as a confounding factor in the cell experiments. These results indicate that VVP-1 and VVP-2 are polysaccharide-rich fractions, with only trace amounts of uronic acid and residual protein.

### 2.3. Molecular-Weight Determination

**Figure 2:**
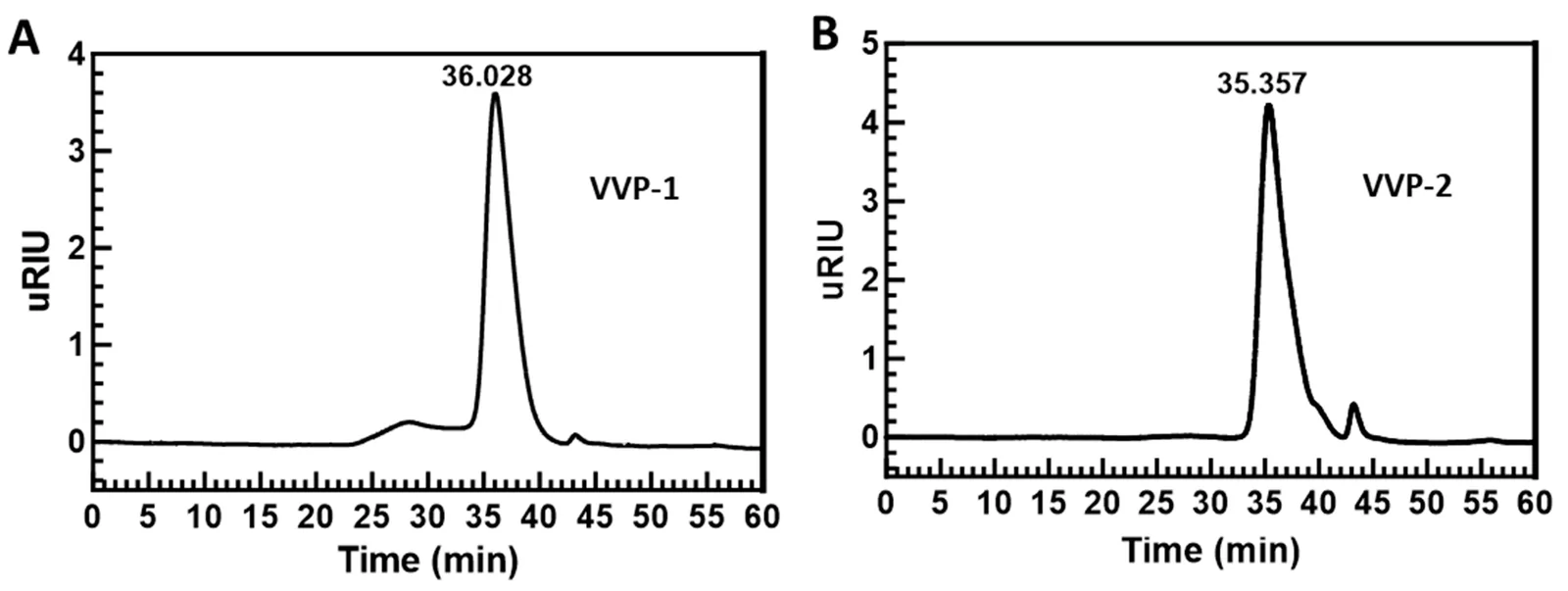
HPGPC chromatograms of VVP-1 and VVP-2. (**A**) VVP-1; (**B**) VVP-2.

The molecular weights of VVP-1 and VVP-2 fractionated by Sephadex G-100 chromatography were measured by HPGPC. As shown in Figure 2A, the principal VVP-1 peak, accounting for more than 85% of the total peak area, had a retention time of 36.028 min. The dextran calibration curve was lg MW = −0.1663x + 10.131 (R^2^ = 0.9949). The principal polysaccharide in VVP-1 had a weight-average molecular weight (Mw) of 13,789 Da, a number-average molecular weight (Mn) of 7528 Da, and an Mw/Mn ratio of 1.83. As shown in Figure 2B, the principal VVP-2 peak, accounting for more than 95% of the total peak area, had a retention time of 35.357 min. VVP-2 had an Mw of 17,829 Da, an Mn of 7542 Da, and an Mw/Mn ratio of 2.36.

### 2.4. Effects of VVP-2 on Lipid Content and Inflammatory Cytokine Expression in PA-Challenged AML12 Cells

**Figure 3:**
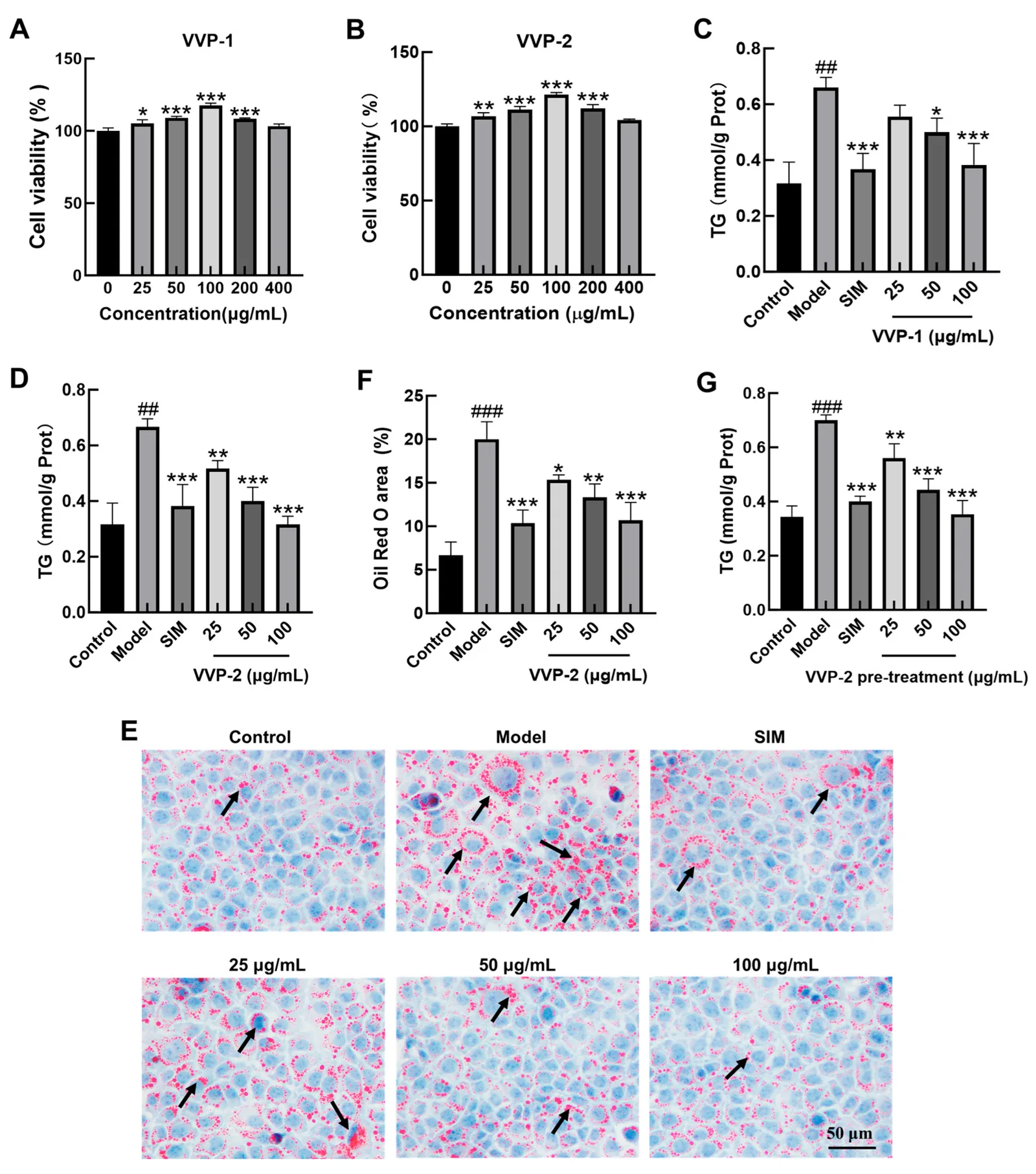
Effects of VVP-2 on lipid accumulation in palmitic acid (PA)-challenged AML12 cells. (**A**,**B**) Effects of VVP-1 and VVP-2 on AML12 cell viability. (**C**,**D**) Intracellular triglyceride (TG) content after co-incubation with indicated concentrations of VVP-1 (**C**) and VVP-2 (**D**). (**E**) Representative Oil Red O staining of intracellular lipid droplets. Black arrows indicate lipid droplets. (**F**) Quantification of Oil Red O-stained areas by ImageJ (1.54p). (**G**) Intracellular TG content after VVP-2 or SIM pretreatment, washout, and PA exposure. Data are presented as the mean ± SD from three independent biological replicates (*n* = 3). For Oil Red O quantification, five random fields per well were analyzed for each biological replicate. ^##^ *p* < 0.01 and ^###^ *p* < 0.001 versus the control group; * *p* < 0.05, ** *p* < 0.01, and *** *p* < 0.001 versus the model group.

AML12 cells were exposed to VVP-1 or VVP-2 at 0, 25, 50, 100, 200, or 400 μg/mL for 24 h, and viability was assessed by the CCK-8 assay. As shown in Figure 3A,B, both polysaccharides increased cell viability in a dose-dependent manner over 0–100 μg/mL, with the highest viability at 100 μg/mL. At 200 and 400 μg/mL, viability declined relative to the 100 μg/mL group but remained at or above the control level. Accordingly, 25, 50, and 100 μg/mL were selected for subsequent experiments.

AML12 cells were co-incubated with 250 μmol/L PA and SIM (20 μM) or VVP-1/VVP-2 (25, 50, or 100 μg/mL) for 24 h. In the model group, intracellular Triglyceride (TG) was significantly higher than in the control group (*p* < 0.01), confirming successful establishment of the high-lipid model. As shown in Figure 3C,D, both polysaccharides reduced the PA-induced increase in TG in a dose-dependent manner. VVP-2 exhibited a trend of greater lipid-lowering effect compared with VVP-1 at the tested concentrations; VVP-2 was therefore selected for mechanistic studies. Oil Red O staining (Figure 3E,F) revealed few lipid droplets in control cells, whereas model cells contained numerous, markedly enlarged red lipid droplets. VVP-2 substantially reduced both lipid droplet size and number relative to the model group, with the greatest effect at the high dose, consistent with the TG assay. To further investigate whether VVP-2 acts primarily by reducing PA uptake, a pre-treatment and washout experiment was performed. As shown in Figure 3G, PA induced a marked increase in intracellular TG in the model group. VVP-2 pretreatment dose-dependently reduced subsequent PA-induced TG accumulation. At 100 μg/mL, TG levels were significantly lower than those in the PA model group and were not significantly different from those in the control group. SIM pretreatment also significantly reduced TG accumulation compared with the PA model group. These results suggest that the effect of VVP-2 is not solely dependent on immediate inhibition of PA uptake during co-incubation and may also involve more persistent intracellular changes that attenuate lipid accumulation.

### 2.5. Effects of VVP-2 on the Transcriptome of PA-Challenged AML12 Cells

**Figure 4:**
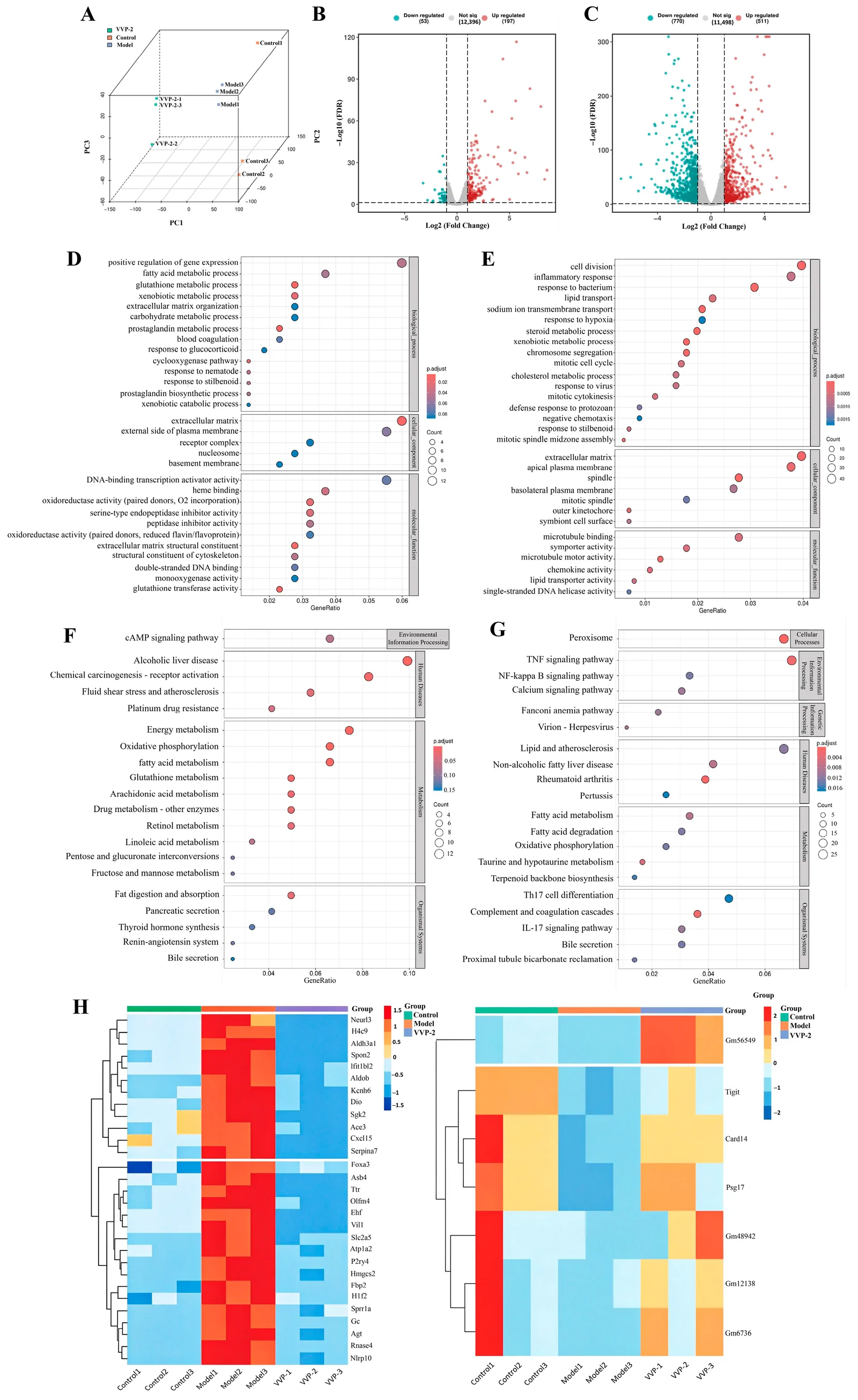
Transcriptome sequencing analysis. (**A**) PCA showing the clustering separation among samples from the blank group, model group, and VVP-2 administration group. (**B**) The number of differentially expressed genes (DEGs) in the model group compared with the blank group. (**C**) The number of DEGs in the administration group compared with the model group. (**D**,**E**) Selected GO terms from enrichment analyses of all DEGs in the model-versus-control and VVP-2-versus-model comparisons, respectively. (**F**,**G**) Selected KEGG pathways from enrichment analyses of all DEGs in the corresponding comparisons. (**H**) Heatmap of genes whose PA-induced changes were partially reversed by VVP-2.

Transcriptome sequencing was performed to further characterize the effects of VVP-2 on PA-challenged AML12 cells. Principal component analysis showed clear separation among the control, model, and VVP-2-treated samples (Figure 4A). Relative to the control group, PA exposure produced 197 upregulated and 53 downregulated genes. Relative to the model group, VVP-2 co-incubation produced 511 upregulated and 770 downregulated genes (Figure 4B,C). GO functional annotation and KEGG pathway enrichment analyses of all differentially expressed genes (DEGs) in each comparison showed that the genes altered by PA were enriched mainly in energy metabolism, oxidative phosphorylation, and lipid metabolism pathways. Following VVP-2 co-incubation, DEGs were concentrated in fatty acid metabolism, fatty acid degradation, mitochondrial oxidative phosphorylation, and inflammation-related pathways (Figure 4D–G). To identify which PA-induced changes were reversed by VVP-2, we compared gene expression trends across the control, model, and VVP-2 groups. A total of 36 genes were partially reversed by VVP-2, most of which were related to metabolic regulation and inflammatory/immune responses. Notably, certain genes such as *Hmgcs2* were regulated by VVP-2 beyond the baseline control levels. These results suggest that VVP-2 may partially reverse PA-induced transcriptomic changes, particularly in genes related to metabolic regulation and inflammatory/immune responses, which may contribute to its lipid-lowering and anti-inflammatory effects. However, these findings are correlative and require further validation.

### 2.6. Effects of VVP-2 on Lipid Metabolism-Related Gene Expression

**Figure 5:**
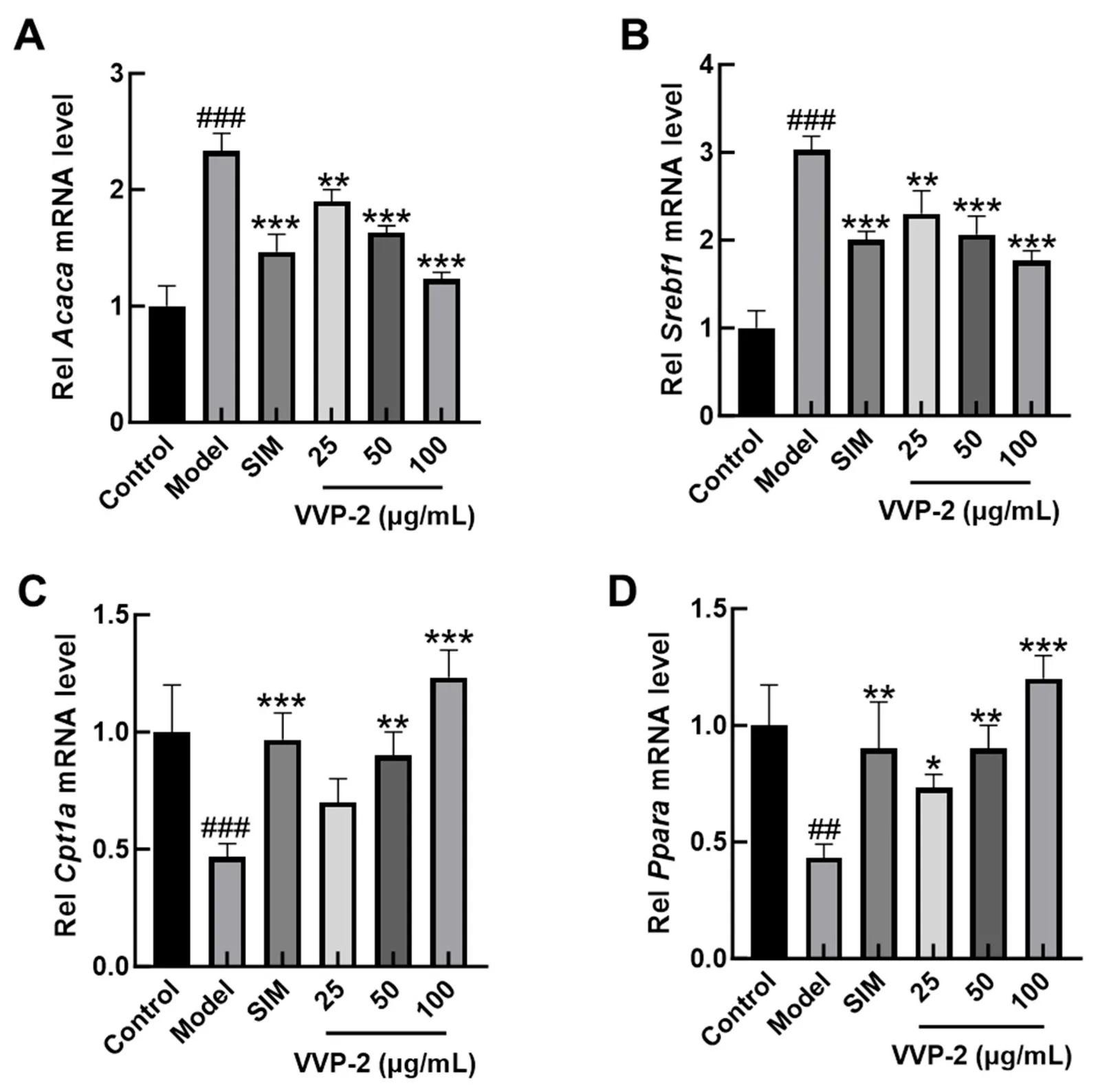
Effects of VVP-2 on the expression of lipid metabolism-related genes. The mRNA levels of acetyl-CoA carboxylase (*Acaca*) (**A**), sterol regulatory element-binding protein 1 (*Srebf1*) (**B**), carnitine palmitoyltransferase1 (*Cpt1a*) (**C**), and peroxisome proliferator-activated receptor alpha (*Ppara*) (**D**) in AML12 cells were detected by RT-qPCR. Data are presented as the mean ± SD from three independent biological replicates (*n* = 3). ^##^ *p* < 0.01 and ^###^ *p* < 0.001 versus the control group; * *p* < 0.05, ** *p* < 0.01, and *** *p* < 0.001 versus the model group.

To further explore potential molecular changes associated with the lipid-lowering effect of VVP-2, the mRNA levels of key lipid-metabolism genes were measured by RT-qPCR. As shown in Figure 5A,B, the lipogenic genes acetyl-CoA carboxylase (*Acaca*) and sterol regulatory element-binding protein 1 (*Srebf1*) were significantly upregulated in the model group relative to the control group. VVP-2 co-incubation was associated with a significant, dose-dependent downregulation of both genes relative to the model group. Conversely, the fatty acid oxidation genes carnitine palmitoyltransferase1 (*Cpt1a*) and peroxisome proliferator-activated receptor alpha (*Ppara*) were markedly downregulated in the model group, whereas VVP-2 co-incubation was associated with a significant, dose-dependent restoration of their expression (Figure 5C,D). A comparison of these RT-qPCR results with the RNA-seq data revealed consistent directional trends for *Acaca*, *Srebf1*, and *Ppara*, but a discrepancy for *Cpt1a* (RNA-seq showed a slight downregulation, whereas RT-qPCR showed an upregulation) (Supplementary Table S1). This inconsistency may be attributed to primer specificity for transcript variants or post-transcriptional regulation.

### 2.7. Effects of VVP-2 on Mitochondrial Function in AML12 Cells

**Figure 6:**
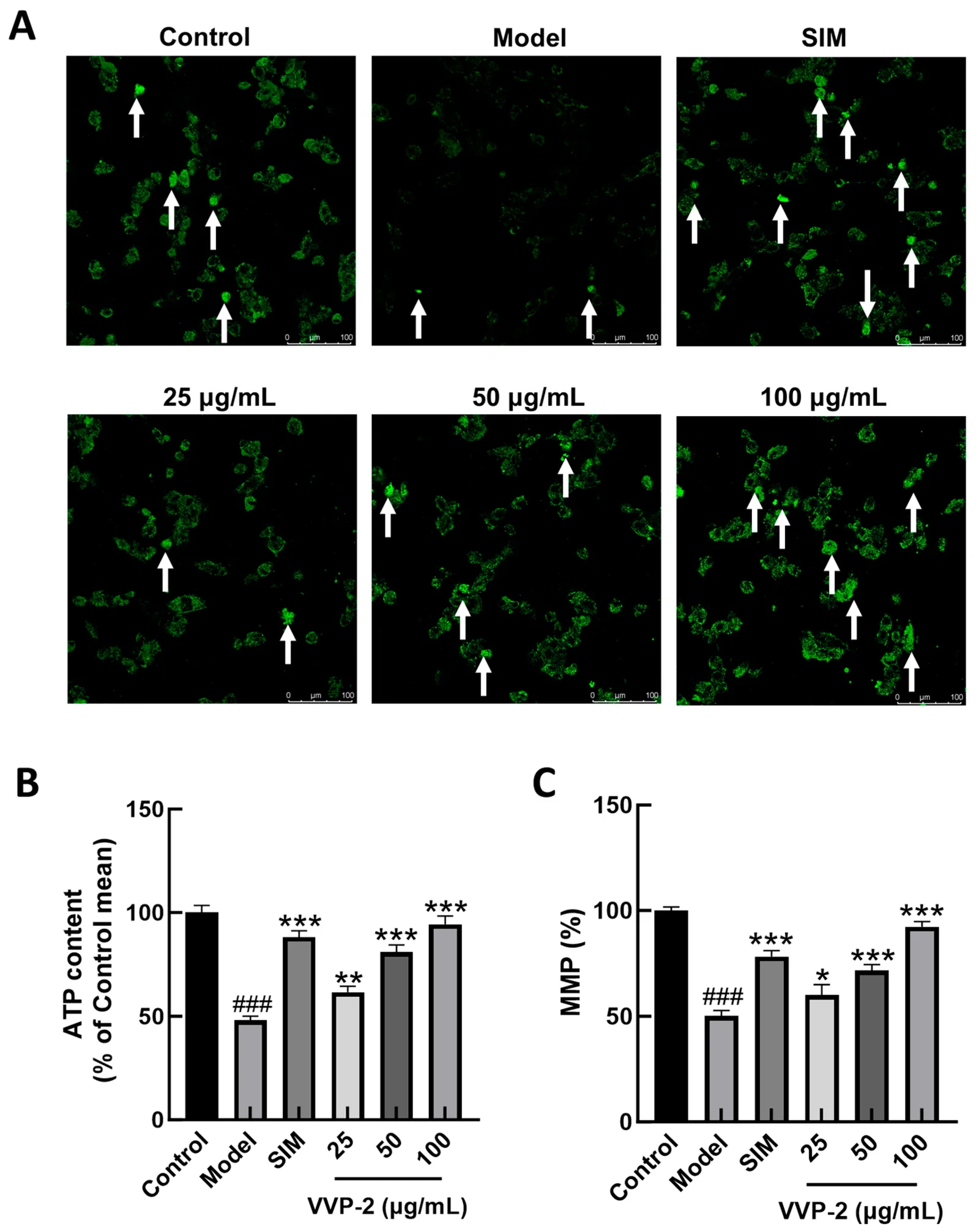
Effects of VVP-2 on mitochondrial function in AML12 cells. (**A**) Representative images of Mito-Tracker Green staining are shown (qualitative assessment). White arrows indicate representative mitochondrial signal. (**B**) Determination of ATP content. (**C**) Measurement of mitochondrial membrane potential. Data are presented as the mean ± SD from three independent biological replicates (*n* = 3). ^###^ *p* < 0.001 versus the control group; * *p* < 0.05, ** *p* < 0.01, and *** *p* < 0.001 versus the model group.

Because mitochondria are the principal site of fatty acid β-oxidation, indices of mitochondrial function were examined [23]. Mito-Tracker Green staining showed visibly weaker green fluorescence in the model group than in the control group. VVP-2 co-incubation appeared to restore the fluorescence intensity relative to the model group (Figure 6A). Intracellular ATP was significantly reduced by PA exposure but was increased by VVP-2 in a dose-dependent manner; the high dose restored ATP to near-control levels (Figure 6B). JC-1 analysis showed that mitochondrial membrane potential was significantly reduced in the model group. VVP-2 co-incubation was associated with a significant restoration of membrane potential, with the largest increase at the high dose (Figure 6C).

Taken together, these results indicate that VVP-2 may improve mitochondrial function. These changes were associated with preservation of mitochondrial signal, enhanced ATP production, and restoration of mitochondrial membrane potential. These changes were accompanied by modulation of lipid metabolism-related gene expression and reduced intracellular lipid accumulation.

### 2.8. Effects of VVP-2 on Inflammatory Responses in AML12 Cells

**Figure 7:**
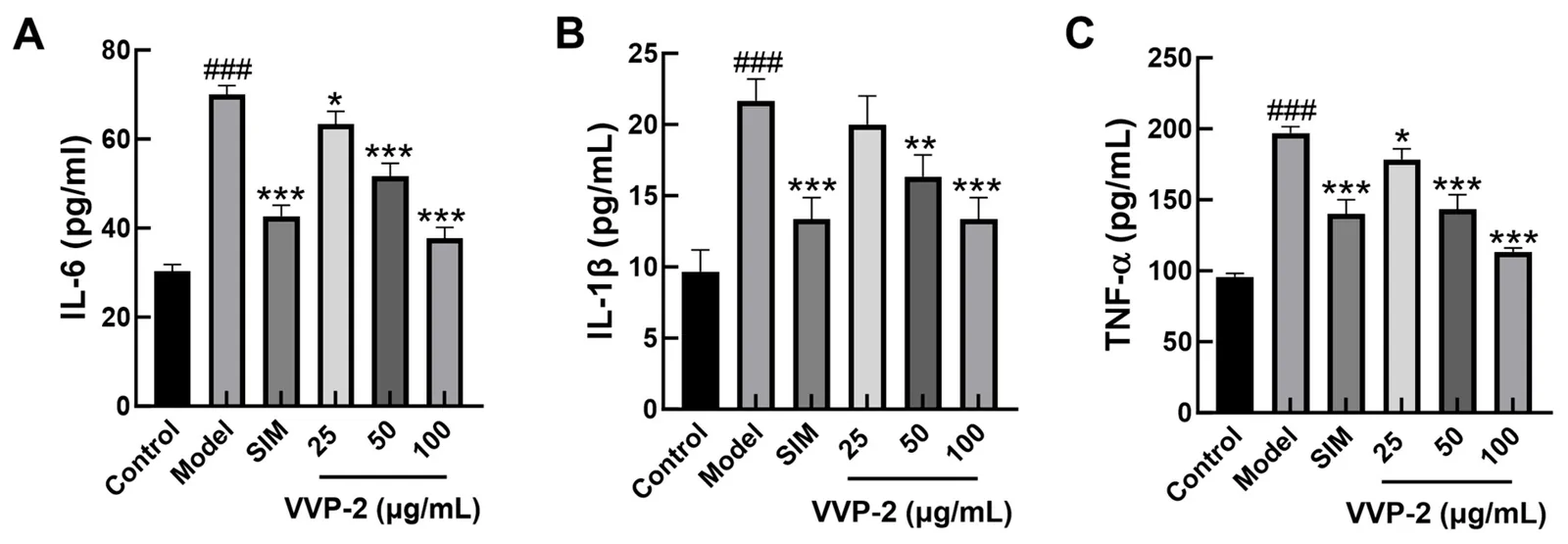
Effects of VVP-2 on inflammatory cytokine production in PA-challenged AML12 cells. Levels of IL-6 (**A**), IL-1β (**B**), and TNF-α (**C**) in the cell culture supernatant were measured by ELISA. Data are presented as the mean ± SD from three independent biological replicates (*n* = 3). ^###^ *p* < 0.001 versus the control group; * *p* < 0.05, ** *p* < 0.01, and *** *p* < 0.001 versus the model group.

Chronic inflammation is an important pathological feature of lipid metabolic dysfunction, and excessive lipid accumulation can activate inflammatory signaling. To assess the regulation of inflammatory responses by VVP-2 under high-lipid conditions, IL-1β, IL-6, and TNF-α levels in cell-culture supernatants were measured by ELISA. As shown in Figure 7A–C, all three cytokines were significantly elevated in the model group relative to the control group, suggesting that PA-induced lipid loading was associated with an inflammatory response in AML12 cells. VVP-2 co-incubation was associated with a dose-dependent reduction in all three cytokines relative to the model group. The strongest inhibition occurred at 100 μg/mL.

### 2.9. Effects of VVP-2 on Whole-Body Metabolism in HFD-Induced Obese Mice

**Figure 8:**
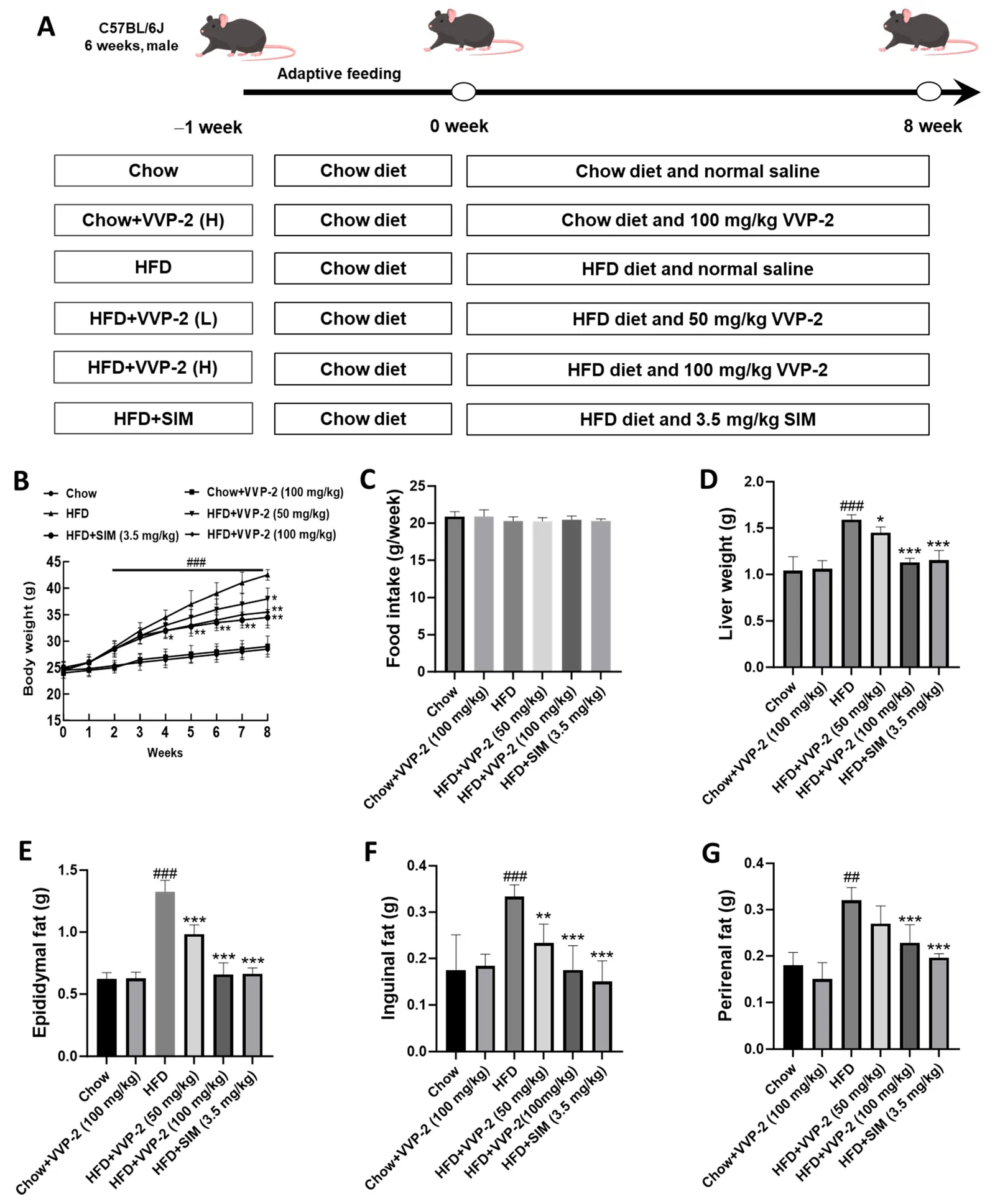
Effects of VVP-2 on lipid metabolism in HFD-fed mice. (**A**) Schematic diagram of animal grouping and drug administration procedure. (**B**) Changes in mouse body weight over time. Data were analyzed by two-way repeated-measures ANOVA (time × group) followed by Tukey’s post hoc test. (**C**) Food intake of mice. (**D**) Liver weight of mice. (**E**–**G**) Epididymal, inguinal, and perirenal fat weights of mice. Data are presented as the mean ± SD (*n* = 6). ^##^ *p* < 0.01, ^###^ *p* < 0.001 versus the chow group; * *p* < 0.05, ** *p* < 0.01, and *** *p* < 0.001 versus the HFD group at the corresponding time point.

SIM at 3.5 mg/kg was used as a positive control to systematically evaluate the ability of VVP-2 to ameliorate HFD-induced metabolic abnormalities. HFD-induced obese mice received VVP-2 at 50 or 100 mg/kg by oral gavage for eight weeks, and body weight was measured weekly. As shown in Figure 8B, HFD-fed mice exhibited progressive weight gain, whereas VVP-2 significantly attenuated this weight gain from week 4 onward (time × group interaction, *p* < 0.001; post hoc *p* < 0.05 versus the HFD group). At week 8, both VVP-2 doses significantly reduced body weight relative to the HFD group (*p* < 0.05; *p* < 0.01). Food intake did not differ significantly among groups, excluding differences in food consumption as the cause of the changes in body weight (Figure 8C). HFD feeding also significantly increased liver, epididymal, inguinal, and perirenal fat weights. Both VVP-2 doses and SIM effectively reduced liver and adipose tissue weights (Figure 8D–G).

### 2.10. Effects of VVP-2 on Hepatic Lipid Accumulation and Liver Injury in HFD-Induced Obese Mice

**Figure 9:**
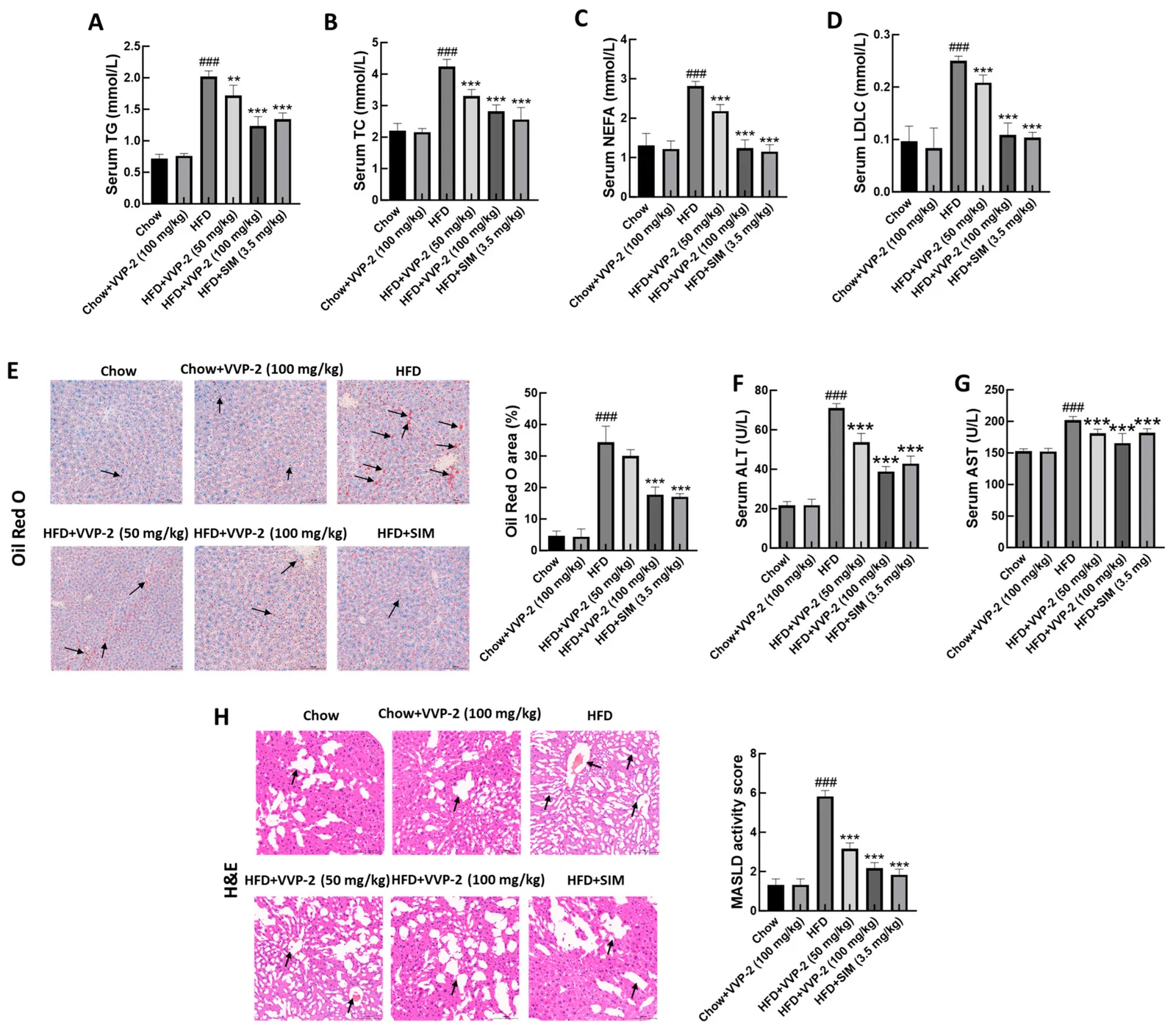
Effects of VVP-2 on hepatic lipid accumulation and liver injury in HFD-fed mice. (**A**–**D**) Determination of serum TG, total cholesterol (TC), non-esterified fatty acids (NEFA), and low-density lipoprotein cholesterol (LDL-C). (**E**) Representative images of hepatic Oil Red O staining and semi-quantitative analysis performed using ImageJ (1.54p). Black arrows indicate lipid droplets. (**F**,**G**) Serum alanine aminotransferase (ALT) (**F**) and aspartate aminotransferase AST (**G**) levels. (**H**) Representative H&E-stained liver sections and MASLD activity scores (MAS). Black arrows indicate pathological changes. Data are presented as the mean ± SD (*n* = 6). ^###^ *p* < 0.001 versus the chow group; ** *p* < 0.01, and *** *p* < 0.001 versus the HFD group.

As shown in Figure 9A–D, serum TG, total cholesterol (TC), non-esterified fatty acids (NEFA), and low-density lipoprotein cholesterol (LDL-C) were significantly higher in the HFD group than in the chow group. All four indices were significantly reduced by low- or high-dose VVP-2 and by SIM. Oil Red O staining revealed severe hepatic lipid deposition in HFD-fed mice, which was markedly alleviated by VVP-2 co-administration with HFD (Figure 9E). Serum alanine aminotransferase (ALT) and aspartate aminotransferase (AST), two sensitive markers of hepatic injury, were also markedly elevated in HFD-fed mice, indicating diet-induced liver damage. Both VVP-2 doses and SIM significantly reduced these enzyme levels relative to the HFD group (Figure 9F,G). H&E staining further showed pathological changes in HFD-fed livers, including pronounced hepatocyte ballooning and inflammatory infiltration. These abnormalities were substantially attenuated by VVP-2, as reflected by lower MASLD activity scores (MAS) values (Figure 9H).

## 3. Discussion

This study successfully isolated two polysaccharide-enriched fractions, VVP-1 and VVP-2, from *V. volvacea* fruiting bodies using hot-water extraction, ethanol precipitation, deproteinization, and two-step column chromatography. Their weight-average molecular weights were 13,789 and 17,829 Da, respectively. In vitro experiments showed that VVP-2 dose-dependently reduced TG and lipid droplet accumulation in PA-challenged AML12 hepatocytes. The observed effects may involve the coordinated modulation of lipid synthesis and fatty acid oxidation-related gene expression, attenuation of inflammation, and improvement of mitochondrial function.

Extraction and fractionation provide the foundation for evaluating polysaccharide bioactivity. TCA deproteinization reduced protein content from 21.0% to 2.5%, equivalent to approximately 88.1% removal, showing that the method effectively removed free protein, consistent with most studies of fungal polysaccharide deproteinization [24]. During DEAE-52 chromatography, crude VVPs were concentrated mainly in the water-eluted and 0.1 mol/L NaCl-eluted fractions, suggesting that neutral and weakly acidic polysaccharides were predominant. After further fractionation on Sephadex G-100, both fractions produced single symmetrical peaks, indicating relatively uniform molecular-weight distributions. The Mw values of VVP-1 and VVP-2 were 13,789 and 17,829 Da, respectively, placing both within the low-to-medium molecular-weight range. Low-molecular-weight polysaccharides generally have better solubility and bioavailability and may interact more readily with molecular targets [25]. Nevertheless, the relationship between molecular weight and lipid-lowering activity is not simply linear and may also be influenced by monosaccharide composition, glycosidic-linkage patterns, branching, and higher-order structure.

Both polysaccharides promoted AML12 cell viability without overt cytotoxicity at 0–100 μg/mL. TG assays and Oil Red O staining consistently showed that VVP-2 appeared to be more effective in ameliorating PA-induced lipid accumulation compared with VVP-1. We hypothesize that this difference in activity may be related to differences in structural or physicochemical properties, such as monosaccharide composition, glycosidic linkages, branching, or molecular conformation. However, these structural features were not determined in the present study. Monosaccharide composition, glycosidic-linkage analysis, infrared spectroscopy, nuclear magnetic resonance, and other structural approaches will be needed to clarify the basis of the difference.

Previous studies have reported that edible mushroom polysaccharides ameliorate lipid metabolic disorders through diverse mechanisms, including modulation of gut microbiota, activation of AMPK/Sirt1/PGC-1α signaling, and regulation of short-chain fatty acid production [12,13,14]. In the present study, VVP-2 co-incubation was associated with improved mitochondrial function, upregulation of fatty acid oxidation-related genes, and reduced inflammatory cytokine secretion in PA-challenged hepatocytes. These findings are consistent with the lipid-lowering effects reported for other fungal polysaccharides, but the present work focused on mitochondrial function and lipid metabolism-related gene expression rather than gut microbiota. In addition, previous work showed that VVPs inhibit fat accumulation in C. elegans via the aak-2/nhr-49-mediated pathway [22]. Our findings extend these observations to a mammalian hepatocyte model and an HFD-induced obese mouse model, suggesting that VVP-2 may ameliorate hepatic lipid accumulation, at least in part, through mechanisms involving mitochondrial function and lipid metabolism-related gene expression. However, direct comparisons across studies are limited by differences in polysaccharide source, molecular weight, dosage, and experimental models.

Hepatic lipid homeostasis is tightly regulated by the balance between lipid synthesis and fatty acid oxidation [26]. ACC is a rate-limiting enzyme in de novo fatty acid synthesis, whereas SREBP-1 is a key transcription factor regulating lipogenic gene expression [27,28]. PPAR-α is a central regulator of fatty acid oxidation and promotes mitochondrial β-oxidation by inducing genes such as *CPT1* [29]. In the present study, PA increased *Acaca* and *Srebf1* mRNA levels while reducing *Cpt1a* and *Ppara* mRNA levels, suggesting a shift toward increased expression of lipogenic genes and reduced expression of fatty acid oxidation-related genes under high-lipid conditions; this pattern was accompanied by substantial TG accumulation. VVP-2 treatment was associated with a partial reversal of these expression changes, suggesting that its lipid-lowering effect may involve, at least in part, modulation of lipid synthesis and fatty acid oxidation-related gene expression. However, gene expression changes alone cannot establish increased metabolic flux through fatty acid oxidation.

Transcriptome sequencing provided global support for this regulatory pattern. Principal component analysis clearly separated the control, model, and VVP-2 co-incubation groups, showing that VVP-2 systematically altered the transcriptomic profile of hepatocytes under high-lipid stress. GO and KEGG analyses indicated that DEGs produced by PA exposure were enriched mainly in energy metabolism, oxidative phosphorylation, and fatty acid metabolism. After VVP-2 treatment, DEGs were likewise concentrated in fatty acid metabolism and degradation, mitochondrial oxidative phosphorylation, and inflammation-related pathways. These findings were highly consistent with the RT-qPCR and ELISA results and support the hypothesis that VVP-2 may protect hepatocytes by modulating lipid metabolism and inflammatory signaling. However, pathway enrichment does not by itself demonstrate changes in pathway activity or establish causality.

Chronic low-grade inflammation is an important pathological feature of MASLD. It is well established that excess lipid accumulation can activate inflammatory pathways such as NF-κB, promoting the secretion of TNF-α, IL-6, and IL-1β and thereby aggravating hepatocyte injury and insulin resistance [30]. All three cytokines were significantly elevated in PA-challenged cells and were reduced dose-dependently by VVP-2, suggesting that anti-inflammatory activity may contribute to its lipid-lowering effect. Nevertheless, causality cannot be established from these data alone.

Mitochondria are the principal site of fatty acid β-oxidation and are also central to cellular energy metabolism and reactive oxygen species production [31]. Hepatocyte mitochondria in MASLD commonly show reduced abundance, abnormal morphology, impaired oxidative phosphorylation, and decreased ATP synthesis [31]. In this study, PA reduced mitochondrial abundance, ATP content, and membrane potential. VVP-2 significantly improved all three indices, suggesting a protective effect on mitochondrial function. However, these endpoints do not directly measure fatty acid oxidation flux or mitochondrial respiratory activity, and further studies are needed to confirm whether mitochondrial function is causally improved.

Although this study evaluated the in vitro lipid-lowering activity of VVP-2, explored potential associated molecular changes, and verified its in vivo activity in HFD-induced obese mice, several limitations remain. First, the proposed mechanism is correlative. Only mRNA levels of four genes and dye-based mitochondrial readouts were measured. Protein levels of p-ACC, SREBP-1, CPT1A, and PPARα were not determined; fatty acid oxidation flux was not directly measured; and no inhibitor experiment was performed. In addition, these markers were not measured in mouse liver, so the in vitro mechanistic findings cannot be directly linked to the in vivo effects. AMPK was also not assessed, although previous work in C. elegans implicated AMPK signaling. Future studies should include protein-level analysis, functional FAO measurements, inhibitor experiments, and liver tissue validation to establish the mechanism. Second, although VVP-1 and VVP-2 contained high total carbohydrate content, low residual protein, and low endotoxin levels, their chemical homogeneity was not fully established. A single symmetrical chromatographic peak does not by itself prove chemical homogeneity. Monosaccharide composition, glycosidic-linkage analysis, FT-IR, and NMR were not determined in this study. Therefore, VVP-1 and VVP-2 should be regarded as polysaccharide-enriched fractions rather than fully characterized purified polysaccharides, and their structure–activity relationship remains to be established. Finally, this study did not examine effects on the gut microbiota, which may play an important role in the lipid-lowering activity of polysaccharides and warrants further investigation.

## 4. Materials and Methods

### 4.1. Materials and Reagents

Dried *V. volvacea* fruiting bodies were purchased from Yunnan Lurongjun Agricultural Technology Co., Ltd. (Kunming, China) and were identified by the supplier based on morphological characteristics.

AML12 cells were purchased from Wuhan Procell Life Science & Technology Co., Ltd. (Wuhan, China). High-glucose DMEM, fetal bovine serum (FBS), and penicillin–streptomycin solution (100×) were obtained from Gibco (Grand Island, NY, USA). A CCK-8 cell proliferation/cytotoxicity assay kit was purchased from LABGIC Technology Co., Ltd. (Beijing, China). ALT, AST, TG, TC, NEFA and LDL-C assay kits were purchased from Nanjing Jiancheng Technology Co., Ltd. (Nanjing, China). SIM was purchased from Shanghai Aladdin Biochemical Technology Co., Ltd. (Shanghai, China). Mouse IL-6, IL-1β, and TNF-α ELISA kits were obtained from Shanghai Enzyme-linked Biotechnology Co., Ltd. (Shanghai, China). Chromogenic LAL Endotoxin Assay Kit, Mito-Tracker Green, Mitochondrial membrane potential assay kit with JC-1, ATP assay kit, and Oil Red O staining solution were purchased from Beyotime Biotechnology Co., Ltd. (Shanghai, China). Phenol, concentrated sulfuric acid, and TCA were of analytical grade and were purchased from Sinopharm Chemical Reagent Co., Ltd. (Shanghai, China). DEAE-52 cellulose was obtained from Hefei Bomei Biotechnology Co., Ltd. (Hefei, China), and Sephadex G-100 was purchased from Shanghai Yuanye Bio-Technology Co., Ltd. (Shanghai, China).

### 4.2. Major Instruments and Equipment

The principal equipment comprised a multimode microplate reader (Agilent Technologies, Santa Clara, CA, USA), a Series 8000 WJ CO_2_ incubator (Thermo Fisher Scientific, Waltham, MA, USA), an inverted fluorescence microscope (Nikon Corporation, Tokyo, Japan), and a Waters 1515 high-performance liquid chromatograph (Waters Corporation, Milford, MA, USA).

### 4.3. Methods

#### 4.3.1. Extraction of Crude Polysaccharides from *Volvariella volvacea*

Dried *V. volvacea* powder was immersed in 80% ethanol at a solid-to-liquid ratio of 1:4 (g/mL) for 24 h to remove lipids. The supernatant was discarded, and the residue was air-dried. The residue was then extracted three times with water at a solid-to-liquid ratio of 1:20 and 90 °C for 3 h per extraction. The extracts were combined and concentrated under reduced pressure. Absolute ethanol was added to a final ethanol concentration of 80% (*v*/*v*), and the mixture was maintained at 4 °C for 24 h. After centrifugation at 3000 r/min for 30 min, the precipitate was collected and freeze-dried to obtain the crude polysaccharide extract. Approximately 45 g of crude polysaccharides was obtained from 500 g of dried mushroom, corresponding to a yield of approximately 9% (*w*/*w*).

#### 4.3.2. Deproteinization of Crude Polysaccharides

Crude *V. volvacea* polysaccharides were deproteinized with an equal volume of 5% TCA for 4 h [24]; after centrifugation, the supernatant was freeze-dried. Protein contents before and after deproteinization were measured using a BCA kit [32] with BSA standards (0–1000 μg/mL). Sample or standard (20 μL) and BCA working reagent (200 μL) were incubated at 37 °C for 30 min, and absorbance was read at 562 nm. Protein concentrations were calculated from the BSA standard curve.

#### 4.3.3. Fractionation of Crude Polysaccharides

Crude polysaccharides were fractionated by DEAE-52 cellulose column chromatography with sequential elution using 0 (water), 0.1, 0.2, and 0.4 mol/L NaCl. The eluates were monitored by the phenol–sulfuric acid method [33], and peak fractions were pooled, dialyzed (MWCO 3.5 kDa), and freeze-dried. The resulting fractions were further fractionated by Sephadex G-100 column chromatography eluted with deionized water. Peak fractions were collected and freeze-dried to obtain polysaccharide-enriched fractions.

#### 4.3.4. Physicochemical Properties Identification

Total carbohydrate content was determined by the phenol–sulfuric acid method using glucose as the standard. Uronic acid content was measured by the m-hydroxydiphenyl method using galacturonic acid as the standard. Residual protein content was determined by the BCA method as described in Section 4.3.2. Endotoxin levels were measured with a Limulus amebocyte lysate assay kit according to the manufacturer’s instructions.

#### 4.3.5. Molecular-Weight Determination

The molecular weights of the Sephadex G-100-fractionated polysaccharides were determined by HPGPC. The chromatographic system comprised a Waters high-performance liquid chromatograph with a Waters refractive-index detector and two polymer-based, water-compatible SEC (GFC) columns (8 × 300 mm) connected in series. The mobile phase was 0.05 mol/L NaCl at 0.50 mL/min; the column temperature was 40 °C and the injection volume was 30 μL. A calibration curve was established using saccharide standards (dextran) with molecular weights ranging from 180 to 1,060,000 Da. Samples were prepared at 5 mg/mL, filtered through a 0.22-μm membrane, and analyzed. Molecular weights and distributions were calculated from retention times and the calibration curve.

#### 4.3.6. AML12 Cell Culture

AML12 mouse hepatocytes were routinely cultured at 37 °C and 5% CO_2_ in high-glucose DMEM supplemented with 10% FBS and 1% penicillin–streptomycin. At 80–90% confluence, cells were detached with 0.25% trypsin-EDTA and passaged every 2–3 days. Log-phase cells were used in subsequent experiments.

#### 4.3.7. Cell Metabolic Viability Assay

The effects of VVP-1 and VVP-2 on AML12 cell metabolic viability were assessed using the CCK-8 assay [34]. Log-phase cells were seeded in 96-well plates at 1 × 10^4^ cells/well in 100 μL and cultured overnight at 37 °C and 5% CO_2_. The medium was replaced with complete medium containing VVP-1 or VVP-2 at 0, 25, 50, 100, 200, or 400 μg/mL. Five technical replicate wells were used per treatment in each independent experiment, and the entire experiment was repeated three times independently (*n* = 3 biological replicates). Cells were incubated for 24 h. Ten microliters of CCK-8 reagent was then added to each well and incubated for 2 h at 37 °C in the dark. Absorbance was measured at 450 nm. Cell viability was calculated as follows:

Cell viability (%) = (OD of treatment group − OD of blank group)/(OD of control group − OD of blank group) × 100%.

#### 4.3.8. Intracellular TG Assay

Log-phase AML12 cells were seeded in 12-well plates at 1 × 10^5^ cells/well and cultured overnight. The groups were control, model, low-, medium-, and high-dose VVP-1 (25, 50, and 100 μg/mL), low-, medium-, and high-dose VVP-2 (25, 50, and 100 μg/mL), and the positive-control SIM group (20 μM) [35]. PA was conjugated to fatty acid-free bovine serum albumin (BSA) to ensure solubility in aqueous medium. Except for the control group, cells were co-treated with 250 μmol/L PA and the indicated concentrations of VVP-1, VVP-2, or SIM for 24 h. The control group received the same final concentration of fatty-acid-free BSA without PA. After the 24-h incubation, the medium was discarded and the cells were washed twice with PBS. RIPA lysis buffer (200 μL/well) was added, and cells were lysed on ice for 30 min and centrifuged at 12,000 r/min for 10 min at 4 °C. TG in the supernatant was measured according to the kit instructions at 550 nm. TG values were normalized to protein content, which was measured by the BCA method.

To examine whether VVP-2 acts primarily by reducing PA uptake, AML12 cells were pretreated with VVP-2 (25, 50, or 100 μg/mL), SIM (20 μM), or vehicle for 24 h, washed three times with PBS to remove free compounds, and then exposed to 250 μmol/L PA (model and treatment groups) or vehicle (control group) for another 24 h. Control and PA model groups underwent the same 24-h pretreatment and washout steps without VVP-2 or SIM. Intracellular TG was measured as described above.

#### 4.3.9. RNA Sequencing and Bioinformatics Analysis

AML12 cells were assigned to control, model, and VVP-2 (100 μg/mL) groups, with three independent biological replicates per group. Total RNA was extracted with TRIzol and assessed for integrity and concentration. mRNA was enriched using poly-T magnetic beads and fragmented, followed by reverse transcription, end repair, A-tailing, adapter ligation, and PCR amplification to construct cDNA libraries. Library quality and concentration were assessed using an Agilent 2100 system and Qubit 2.0. Qualified libraries with an effective concentration greater than 1.5 nM underwent paired-end 150-bp sequencing on an Illumina platform, yielding an average of 56.1 million clean reads per sample (range 39.0–73.1 million). After quality filtering with fastp v0.23.4, reads were assessed with FastQC v0.11.9, and Q30 > 89.9% was observed across all samples. Clean reads were aligned to the mouse reference genome (GRCm39/mm39) using HISAT2 v2.2.1, with an average total mapping rate of 97.1% (range 94.1–98.1%) and an average unique mapping rate of 90.6% (range 87.6–91.8%). Genes were quantified with featureCounts v2.0.3, and raw counts were normalized using TMM normalization in edgeR v3.28.1. Differentially expressed genes (DEGs) were identified using edgeR v3.28.1 with |log_2_FC| ≥ 1 and FDR < 0.05 (Benjamini–Hochberg correction) for MOD versus NOR and VVP-2 versus MOD. All DEGs meeting the criteria of |log_2_FC| ≥ 1 and FDR < 0.05 in each comparison were included in GO and KEGG enrichment analyses using clusterProfiler v4.2.2. Enrichment was assessed using the hypergeometric test, and *p* values were adjusted using the Benjamini–Hochberg method. GO terms and KEGG pathways with adjusted *p* < 0.05 were considered significantly enriched. To identify genes whose PA-induced changes were potentially reversed by VVP-2, we selected genes that exhibited opposite directional trends (log2FC) between the MOD versus NOR and VVP-2 versus MOD comparisons, with a fold change threshold of >1.5 in at least one comparison.

#### 4.3.10. RT-qPCR Analysis of Lipid Metabolism-Related Gene Expression

Lipid metabolism-related genes were quantified by RT-qPCR [36]. Log-phase AML12 cells were seeded in six-well plates at 2 × 10^5^ cells/well and treated as described in Section 4.3.8. Total RNA was extracted with TRIzol, and its concentration and purity were measured with a NanoDrop 2000 spectrophotometer (Thermo Scientific, Wilmington, DE, USA); A_260_/A_280_ ratios of 1.8–2.0 were accepted. One microgram of total RNA was reverse-transcribed to cDNA according to the kit instructions. Each 20-μL qPCR reaction contained 10 μL SYBR Green premix, 0.8 μL each of forward and reverse primers (10 μmol/L), 2 μL cDNA, and RNase-free water. Cycling conditions were 95 °C for 30 s, followed by 40 cycles of 95 °C for 5 s and 60 °C for 30 s. Melt-curve analysis was used to verify amplification specificity. *Gapdh* was selected as the reference gene because it has been widely validated as a stable internal control in AML12 mouse hepatocytes and in PA–induced hepatic steatosis models [37,38,39], and the relative expression of *Acaca*, *Srebf1*, *Cpt1a*, and *Ppara* was calculated using the 2^−ΔΔCt^ method. Primer sequences are listed in Table 1.

#### 4.3.11. Measurement of Inflammatory Cytokines

Cells were treated in 12-well plates as described in Section 4.3.8. After the 24-h incubation, culture supernatants were collected and centrifuged at 1000 r/min for 5 min at 4 °C. TNF-α, IL-6, and IL-1β were measured in the supernatants using mouse ELISA kits according to the manufacturers’ instructions.

#### 4.3.12. Oil Red O Staining

Oil Red O staining was used to evaluate cellular lipid droplet size and abundance [38]. Log-phase AML12 cells were seeded at 2 × 10^4^ cells/well in 24-well plates containing coverslips and treated as described in Section 4.3.8. Cells were washed twice with PBS, fixed with 4% paraformaldehyde for 15 min, and washed twice again. Fresh Oil Red O working solution was added for 30 min at room temperature in the dark. After brief differentiation with 60% isopropanol, cells were rinsed three times with distilled water, counterstained with hematoxylin for 1 min, blued under running tap water, mounted, and imaged under an inverted microscope. Oil Red O-positive areas were semiquantified using ImageJ (1.54p).

#### 4.3.13. Mito-Tracker Fluorescence Staining

Mitochondrial abundance was assessed using Mito-Tracker Green [40]. After the 24-h incubation as described in Section 4.3.8, cells were washed twice with PBS and incubated with serum-free DMEM containing 100 nmol/L Mito-Tracker Green for 30 min at 37 °C in the dark. Cells were washed three times with PBS and imaged by fluorescence microscopy at excitation and emission wavelengths of 488 and 520 nm, respectively.

#### 4.3.14. Mitochondrial Membrane Potential

Changes in mitochondrial membrane potential were measured using the JC-1 probe [41]. Cells were treated in 12-well plates as described in Section 4.3.8, washed twice with PBS, and incubated in serum-free DMEM containing 5 μg/mL JC-1 for 20 min at 37 °C in the dark. After two PBS washes, 200 μL JC-1 lysis buffer was added per well, and the plate was shaken for 5 min at room temperature in the dark. Lysates were transferred to 96-well plates. Red fluorescence was measured at 525/590 nm excitation/emission and green fluorescence at 490/530 nm. Mitochondrial membrane potential was expressed as the red-to-green fluorescence intensity ratio.

#### 4.3.15. ATP Assay

Cells were treated in 12-well plates as described in Section 4.3.8 and washed twice with PBS. ATP lysis buffer (200 μL/well) was added for 5 min on ice. Lysates were centrifuged at 12,000 r/min for 5 min at 4 °C, and the supernatants were collected. Standards or samples were mixed with ATP detection working solution according to the kit instructions and immediately transferred to white 96-well plates for measurement of relative luminescence units with a microplate reader equipped with a chemiluminescence module. ATP content was calculated from the standard curve and normalized to milligrams of protein.

#### 4.3.16. Animal Grouping and Administration

Male C57BL/6J mice (6 weeks old, body weight 18–20 g) were purchased from Shanghai SLAC Laboratory Animal Co., Ltd. (Shanghai, China). All animal procedures were performed in accordance with the Guidelines for the Care and Use of Laboratory Animals of Zhejiang Chinese Medical University and were approved by the Animal Ethics Committee of Zhejiang Chinese Medical University (approval no. CN20250004). Mice were housed under specific-pathogen-free conditions at 22 ± 2 °C and 55 ± 10% humidity under a 12-h light/dark cycle. After a one-week acclimation period with ad libitum access to standard chow, the mice were randomly allocated to six groups (*n* = 6 per group): chow, chow plus VVP-2 (100 mg/kg), HFD, HFD + VVP-2 (50 mg/kg), HFD + VVP-2 (100 mg/kg), and HFD + SIM (3.5 mg/kg). The sample size (*n* = 6 per group) was determined by a power analysis using G*Power 3.1. Based on our preliminary data (mean serum TG of 2.08 mmol/L in the HFD group and 1.83 mmol/L in the VVP-2 group, with a pooled SD of 0.20), the calculated effect size was f = 0.65. With an α level of 0.05 and a power of 0.80, the required minimum sample size was calculated as 5 mice per group. Therefore, *n* = 6 was used to account for potential attrition. Mice were randomized using a random number table and stratified by initial body weight. All outcome assessments, including body weight, organ weights, and serum biochemistry, were performed by investigators blinded to group allocation. Histopathological scoring was performed in a blinded manner as described below. Predefined exclusion criteria were signs of severe illness or abnormal weight loss. No animals met these criteria, and no animals were excluded from the final analysis. The chow and chow plus VVP-2 groups received standard chow for eight weeks; the remaining four groups received a high-fat diet (HFD, D12492, Research Diets, with 60% kcal from fat, 20% from protein, and 20% from carbohydrate) for eight weeks. Groups receiving VVP-2 or SIM were administered the corresponding agent (10 mL/kg) once daily by oral gavage throughout the eight weeks, whereas the chow and HFD groups received an equal volume of normal saline. SIM at 3.5 mg/kg served as the positive control [42]. Body weight and food intake were measured weekly. At the end of the study, mice were fasted for 12 h (with free access to water), then euthanized by intraperitoneal injection of sodium pentobarbital (200 mg/kg). Blood and tissues were collected immediately. After euthanasia, the liver and individual adipose depots were dissected and weighed.

#### 4.3.17. H&E and Oil Red O Staining

Liver tissues were collected after euthanasia and fixed in 4% paraformaldehyde. Following dehydration, paraffin embedding, and sectioning, tissues were stained with hematoxylin and eosin (H&E) for histopathological analysis. MAS were evaluated in a blinded manner from the H&E-stained sections. The score semiquantitatively assessed hepatic steatosis (0–3), lobular inflammation (0–3), and hepatocyte ballooning (0–2), and the total MAS was the sum of these three components. Fresh liver tissues were also embedded in optimal cutting temperature compound, frozen, sectioned, and stained with Oil Red O to assess hepatic lipid deposition.

### 4.4. Statistical Analysis

Results are expressed as the mean ± standard deviation (mean ± SD). Statistical analyses were performed using GraphPad Prism 6.0. Comparisons between two groups were performed using Student’s *t*-test. Multiple groups were compared by one-way analysis of variance followed by Tukey’s post hoc test for pairwise comparisons. Body weight was analyzed using two-way repeated-measures ANOVA (time × group) followed by Tukey’s post hoc test. *p* < 0.05 was considered statistically significant.

## 5. Conclusions

Two polysaccharide-enriched fractions, VVP-1 and VVP-2, were isolated and fractionated from *V. volvacea*. VVP-2 exhibited a trend of greater activity against PA-induced lipid accumulation in hepatocytes. Transcriptome sequencing suggested that its lipid-lowering action may be associated with the regulation of fatty acid metabolism, mitochondrial oxidative phosphorylation, and inflammation-related pathways. VVP-2 exposure was associated with the downregulation of the lipogenic genes *Acaca* and *Srebf1*, the upregulation of the fatty acid oxidation genes *Cpt1a* and *Ppara*, reduced TNF-α, IL-6, and IL-1β secretion, preservation of mitochondrial signal, the restoration of mitochondrial membrane potential, and enhanced ATP production. These coordinated changes were accompanied by amelioration of lipid metabolic dysfunction in hepatocytes under high-lipid stress. In mice, VVP-2 also attenuated HFD-induced weight gain, reduced liver and adipose tissue weights, improved serum lipid profiles, and alleviated hepatic lipid deposition and liver injury. These findings provide preliminary experimental support for developing *V. volvacea* polysaccharides as lipid-lowering functional food ingredients.

## Figures and Tables

**Table 1:** RT-qPCR primer sequences.

Gene	Forward Primer (5′ → 3′)	Reverse Primer (5′ → 3′)
*Acaca*	GTTCTGTTGGACAACGCCTTCAC	GGAGTCACAGAAGCAGCCCATT
*Srebf1*	CGACTACATCCGCTTCTTGCAG	CCTCCATAGACACATCTGTGCC
*Cpt1a*	TCCAGTTGGCTTATCGTGGTG	TCCAGAGTCCGATTGATTTTTGC
*Ppara*	ATGGTGGACACGGAAAGCC	CGATGGATTGCGAAATCTCTTGG
*Gapdh*	CATCACTGCCACCCAGAAGACTG	ATGCCAGTGAGCTTCCCGTTCAG

## Data Availability

The original contributions presented in this study are included in the article and its Supplementary Material. The raw RNA-seq data (FASTQ files) have been submitted to the NCBI Gene Expression Omnibus (GEO) database. The submission is currently under review by NCBI, and the formal accession number is pending. Further inquiries can be directed to the corresponding authors.

## Supplementary Materials

The following supporting information can be downloaded at: https://www.mdpi.com/article/10.3390/ijms27219750/s1.
