# Supplementary material for: Fractionation of Bioactive Polysaccharides from Volvariella volvacea and Their Ameliorative Effects on Hepatic Lipid Accumulation
Source: Int J Mol Sci. 2026 Oct 30;27(21):9750. doi: 10.3390/ijms27219750 (PMC13762479; doi:10.3390/ijms27219750)
Supplement: Supplementary file 1 [file ijms-27-09750-s001.zip › ijms-4628601-supplementary.pdf]

Table S1. Comparison of RNA-seq and RT-qPCR results for key lipid metabolism genes.

Gene	RNA-seq log2FC (VVP-2 vs MOD)	RNA-seq FDR	RT-qPCR Trend
Acaca	-0.27	1.86E-04	Down
Srebf1	-0.51	1.45E-13	Down
Cpt1a	-0.56	1.57E-15	Up (Discrepancy)
Ppara	+0.29	0.032	Up
